# 

**Published:** 1898-03

**Authors:** 


### CONTENTS.

ORIGINAL ARTICLES: PAGE
A Further Contribution to the Hygiene of Milk. By DR. OTT . . . . . 133
Arecolin in Colic. By S. J. J. HARGER, V.M.D. . . . . . . . . . 140
A Review of Our Knowledge of Phyto-Bezoars. By J. W. HARSHBERGER, PH.D. . 143
Traumatic Pericarditis of the Ox. By R. C. MOORE, D.V.S. . . . . . . 146
ABSTRACTS FROM FOREIGN JOURNALS:
Contribution to the Etiology of Lameness in Colts . . . . . . . . 150
Empyema of the Cavity of the Right Upper Jawbone in the Horse . . . . 156
REPORTS OF CASES:
Some Cases in Cattle-practice. Selected by R. H. HARRISON, D.V.S. . . . 158
Clinical Reports—Veterinary Hospital, University of Pennsylvania. Clinic of S. J. J. HARGER, V.M.D. . . . . . . . . . . . . . 165
EDITORIALS:
A Contemplated Blow at Our Horse-industry . . . . . . . . . 168
Leads Veterinary Journalism in America . . . . . . . . . . 168
State Work Well Done . . . . . . . . . . . . . . 169
NECROLOGY . . . . . . . . . . . . . . . . . 171
REVIEW . . . . . . . . . . . . . . . . . . 172
CONTROL WORK:
Hawaiian Laws for the Government of Cattle-Traffic . . . . . . . 175
Report of the Veterinary Department of the Minnesota State Board of Health . 180
SOCIETY PROCEEDINGS:
Chicago Veterinary Society—Iowa State Veterinary Medical Association—Oneida County Veterinary Medical Association—Veterinary Medical Association of New York County—Veterinary Medical Society of the Ontario Veterinary College—Michigan State Veterinary Medical Association—Keystone Veterinary Medical Association—Pennsylvania: Appeal for Greater Interest in the U. S. V. M. A.—Ohio State Veterinary Medical Association—Veterinary Medical Society, University of Pennsylvania—U. S. V. M. A.—Pennsylvania State Veterinary Medical Association . . . . . . . . . . . . . . . . 189
PERSONALS . . . . . . . . . . . . . . . . . 210
NEW INVENTIONS . . . . . . . . . . . . . . . 213

